# Empowerment in Adolescent Patients with a Disability/Chronic Condition: A Scoping Review

**DOI:** 10.3390/children12010049

**Published:** 2024-12-31

**Authors:** Kennedy Austin, Carly Pistawka, Colin J. D. Ross, Kathryn A. Selby, Alice Virani, Vanessa Kitchin, Alison M. Elliott

**Affiliations:** 1 Department of Medical Genetics, University of British Columbia, Vancouver, BC V6T 1Z3, Canada; kaustin5@student.ubc.ca (K.A.); colin.ross@ubc.ca (C.J.D.R.); alice.virani@cw.bc.ca (A.V.); 2 BC Children’s Hospital Research Institute, Vancouver, BC V5Z 4H4, Canada; carly.pistawka1@bcchr.ca (C.P.); kselby@cw.bc.ca (K.A.S.); 3 Centre for Advancing Health Outcomes, St. Paul’s Hospital, Vancouver, BC V6Z 1Y6, Canada; 4 Faculty of Pharmaceutical Sciences, University of British Columbia (UBC), Vancouver, BC V6T 1Z3, Canada; 5 Department of Pediatrics, University of British Columbia, Vancouver, BC V6H 3V4, Canada; 6 Ethics Service, Provincial Health Services Authority, Vancouver, BC V6H 4C1, Canada; 7 Woodward Library, University of British Columbia, Vancouver, BC V6T 1Z3, Canada; vanessa.kitchin@ubc.ca; 8 Women’s Health Research Institute, Vancouver, BC V6H 3N1, Canada

**Keywords:** empowerment, adolescents, disability, chronic condition

## Abstract

Background/Objectives: Empowerment has been associated with several positive outcomes in healthcare; however, there is limited insight on empowerment levels within the adolescent population of those with a chronic condition/disability. The aim of this scoping review was to identify gaps in the existing literature on empowerment levels within this population. Methods: Five databases (MEDLINE [Ovid], EMBASE [Ovid], PsycINFO [Ebsco], CINHAL [Ebsco] and Web of Science [UBC]) and grey literature were searched. Results: A total of 67 studies were included and used for data extraction including descriptive numerical analysis followed by a narrative review. Extracted data were divided into demographic characteristics (e.g., ethnicity/ancestry), type of disability/condition (e.g., type 1 diabetes), interventions used to increase empowerment or empowerment-adjacent elements, quantitative and qualitative tools used to measure empowerment (e.g., questionnaires and/or interviews), domains/outcomes associated with empowerment (e.g., self-control), and review articles. Several interventions were shown to have positive effects on empowerment levels in adolescents with a chronic condition/disability. Conclusions: Gaps were identified in the consideration of ethnicity/ancestry and socioeconomic status, demonstrating a need for future research in this space to focus on the intersection of disability, ethnicity/ancestry, and socio-economic status and the implementation of interventions promoting empowerment.

## 1. Introduction

The period of adolescence, defined by the World Health Organization as ages 10–19, is a critical stage in development that is accompanied by identity changes, often fuelled through life events such as living with a chronic condition or disability [1]. With the continuous growth of this population, it is important to understand their distinct needs and work to improve adolescent health-related outcomes [2]. One key health-related outcome being studied in the adolescent population is empowerment [3,4].

Empowerment has numerous and varied definitions, all of which were considered for this scoping review [5,6]. Some recurring themes of empowerment include a sense of self-control, self-efficacy, self-determination, and self-confidence, attributed with high levels of self-esteem and a positive self-concept [6,7,8]. Health-related empowerment involves feelings of control over health-related matters and the ability to manage one’s own disability [9]. Additionally, providing patients with increased knowledge and skills related to their condition is associated with high levels of empowerment [5], and it has been found that supporting adolescents’ empowerment promotes positive development [10].

While empowerment has been observed as an important aspect in patient healthcare, there are limited studies regarding adolescent-perceived empowerment levels related to disability. Previous research has demonstrated an association between increased reports of empowerment in patients and an improvement in clinical outcomes, pain management, treatment satisfaction, and positive emotions [11]. Most studies, however, have focused primarily on adult populations with chronic conditions such as cancer and diabetes. In both disorders, it was shown that patients with increased levels of empowerment have a greater sense of self-control related to their diagnosis [12,13,14]. There is, however, a lack of understanding regarding levels of empowerment within the adolescent population of those with a chronic condition or disability.

The term “disability’’ is an umbrella term encompassing the impairments resulting from an injury or disease, as well as the physical and/or emotional functional limitations one develops as a result of impairments [15]. Disability also refers to, and recognizes the impact on, an individual caused by apparent environmental restrictions or exclusions present due to one’s impairments [15]. This scoping review includes apparent (e.g., muscular dystrophy) and non-apparent disabilities (e.g., intellectual disability), as well as genetic and non-genetic chronic health conditions. This was done in an attempt to gain a broad overview and understanding of empowerment in adolescents with a variety of disabilities.

The objective of this scoping review was to provide an overview of the existing literature on empowerment levels in adolescent patients with a chronic condition or disability.

## 2. Methods

This scoping review followed the methodological framework described by Arksey and O’Malley [16] which includes identifying a research question followed by identifying and selecting relevant studies, charting the data and reporting the findings, along with refinements proposed to this framework by Colquhoun et al. [17] (recommendations of a consistent label and definition of the term “scoping review”), as well as the PRISMA-ScR statement [18]. The protocol was registered on OSF (OSF | Scoping Review Protocol- Kennedy Austin.docx, available online: https://osf.io/ur2hm; accessed on 14 November 2023).

### 2.1. Search Strategy

The search strategy aimed to identify published articles, unpublished studies, and grey literature. The search strategy was designed in collaboration with a UBC health sciences librarian (VK) and subject expert (AME) and underwent a Peer Review of Electronic Search Strategy (PRESS) by a secondary research librarian. Five databases were used for the search: MEDLINE (OVID), EMBASE (OVID), PsycINFO (EBSCO), CINHAL (EBSCO), and Web of Science (University of British Columbia Institutional Access). Chronic disease/disorder/condition, disability, empower/empowerment, pediatrics, adolescent, child, young adult, and youth were used as key search terms for this scoping review. The full search strategies are available in Supplementary Materials (Supplementary Table S1A). Supplemental searches of grey literature included the first 30 pages of the sites found via Google Scholar as recommended by Haddaway et al. [19]

### 2.2. Study Selection

Studies were screened in Covidence in a two-step process. The initial screening process involved title and abstract screening, followed by a secondary full-text screening of articles. Studies excluded in the secondary full-text screening were recorded with reason for exclusion. Duplicate studies were both automatically removed via Covidence and manually removed by the reviewers. All articles were screened by the first author (KA) and second author (CP). Any conflicts between the reviewers were resolved through discussion, or with an additional reviewer (AME).

Studies that included/covered/reviewed/analyzed the evaluation of patient empowerment in the adolescent population of those with a chronic condition or disability were included in the scoping review. Adolescents were considered between the ages of 10 and 19, as defined by the World Health Organization [20]. Articles that included the perspective of adolescents with any chronic condition or disability (both apparent and non-apparent) were included. The study design was not restricted in an attempt to map the literature and identify knowledge gaps in these settings. Studies including data of participants both within and outside of the 10–19 age range were included if data within the age range could be clearly identified. Studies measuring/evaluating empowerment were included. Studies which developed or validated tools for measuring empowerment were included only if implementation of the tool(s) and data analysis occurred within the adolescent population. Studies measuring outcomes adjacent to empowerment (e.g., quality of life, shared decision making, etc.) were excluded if empowerment was not also measured as an outcome. Studies measuring outcomes related to transition-readiness or transition in healthcare were excluded if empowerment was not also measured as an outcome. Studies were limited to English language. We included studies with publication types including peer-reviewed journal articles, dissertations, and grey literature. Studies in the form of conferences, posters, or meeting abstracts were excluded. Studies that were not accessible through the UBC library database were excluded during full-text screening. The search was ended on October 25th, 2023; all studies published before this date were screened.

### 2.3. Data Extraction

Data were extracted from articles that met the inclusion criteria and were included in the scoping review. The extracted data included recorded information with specific details about the first author, title, year of publication, journal name, type of publication, country of publication, age range, ethnicity/ancestry, socioeconomic status, gender/sex of participants, type of disability/condition, intervention used in population, study design, qualitative methods, quantitative methods, use of control group, objectives, and empowerment-related outcomes (Supplementary Table S1B). Descriptive analysis was carried out on variables of interest and presented in multiple figures and tabular forms to organize the extracted data. A narrative summary was included in this scoping review to further explore generated themes and to provide information regarding gaps in the evaluation of empowerment in adolescent patients with a chronic condition or disability. Extracted data were divided into (1) descriptive numerical analysis and demographic characteristics (i.e., age range, country of publication, ethnicity/ancestry, socioeconomic status, and sex/gender), (2) type of disability/condition (e.g., type 1 diabetes, neurodevelopmental, neuromuscular, autoimmune, etc.), (3) interventions used within the population to increase empowerment or empowerment-adjacent elements, (4) quantitative and qualitative tools used to measure empowerment (e.g., questionnaires and/or interviews), (5) domains and outcomes associated with empowerment, and (6) review articles.

## 3. Results

**Figure 1:**
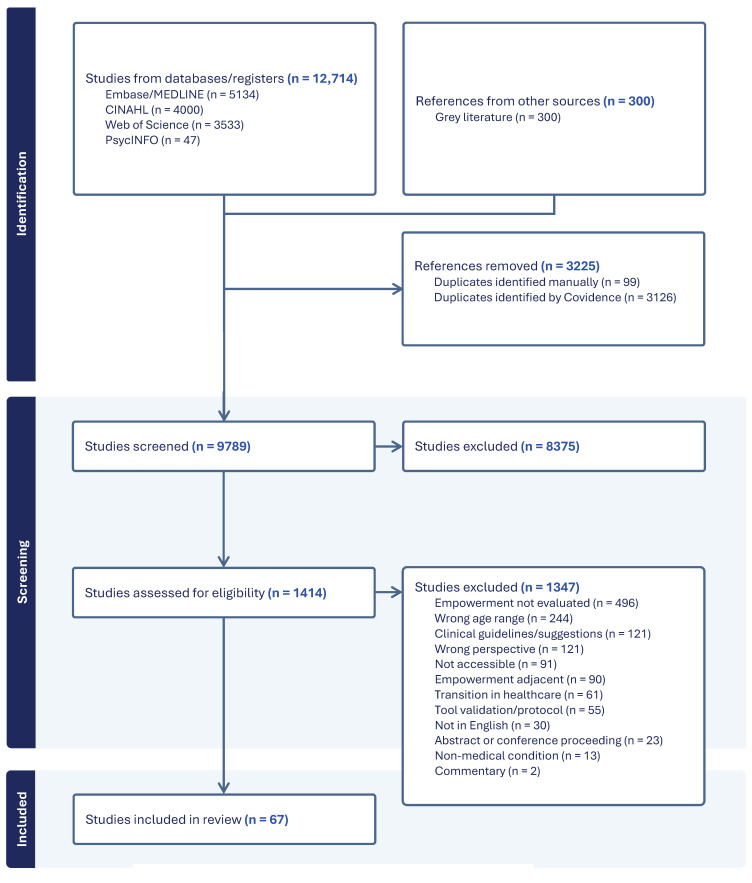
PRISMA flow diagram of study selection process.

The search across the databases and grey literature yielded 13,014 citations (*n* = 12,714 from databases, *n* = 300 from grey literature). A total of 3225 references were excluded as duplicates (3126 identified by Covidence and 99 identified manually by the reviewers). Initial screening of 9789 title and abstracts resulted in the exclusion of 8375 studies not meeting the inclusion criteria. A total of 1414 studies were retrieved for full-text screening where 1347 studies were excluded for the following reasons: empowerment not evaluated (*n* = 496), incorrect age range (*n* = 244), clinical guidelines only (*n* = 121), wrong perspective (e.g., parents, healthcare providers) (*n* = 121), not accessible through UBC library (*n* = 91), empowerment-adjacent (*n* = 90), transition in healthcare (*n* = 61), tool validation/protocol (*n* = 55), not in English (*n* = 30), abstract or conference proceeding (*n* = 23), non-medical condition (*n* = 13), and commentary (*n* = 2). A total of 67 articles were included in the final review [3,21,22,23,24,25,26,27,28,29,30,31,32,33,34,35,36,37,38,39,40,41,42,43,44,45,46,47,48,49,50,51,52,53,54,55,56,57,58,59,60,61,62,63,64,65,66,67,68,69,70,71,72,73,74,75,76,77,78,79,80,81,82,83,84,85,86] (alphabetically ordered based on author in Supplementary Table S2A,B). A PRISMA flow diagram is included which tracks the number of studies included and excluded in the review (Figure 1).

### 3.1. Descriptive Numerical Analysis and Demographics

**Figure 2:**
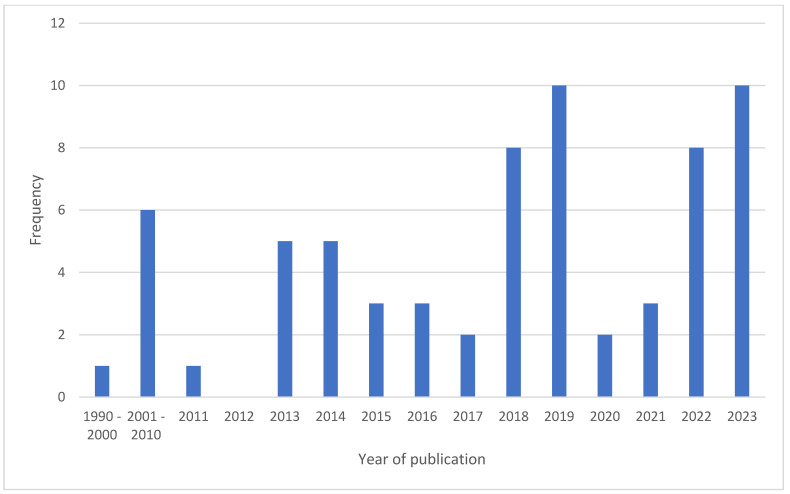
Studies by year of publication.

Of the 67 included studies, one study was published between 1990 and 2000, six were published between 2001 and 2010, 14 were published between 2011 and 2015, 25 were published between 2016 and 2020, and 21 were published between 2021 and October of 2023 (Figure 2).

Demographic characteristics were extracted from the original research studies (*n* = 61) [21,22,23,24,25,26,27,28,29,30,31,32,33,34,35,36,37,38,39,40,41,42,43,44,45,46,47,48,49,50,51,52,53,54,55,56,57,58,59,60,61,62,63,64,65,66,67,68,69,70,71,72,73,74,75,76,77,78,79,80,81]. Data from the included review articles (*n* = 6) [3,82,83,84,85,86] were analyzed separately. The extracted demographic data included age range of participants, country of publication, ethnicity/ancestry, socioeconomic status, and gender/sex of participants.

### 3.2. Age Range of Participants

This scoping review included studies with an adolescent (ages 10–19 years) perspective. Of the 61 original research articles included in this review, 59 provided a participant age range, while two studies did not provide an age range and only included the mean age of participants [24,39]. The youngest age reported was from one study with an age range of 5–18 years [52], while the oldest age reported was from a study with an age range of 10–52 years [48]. Both studies included separate results related to empowerment of individuals within the 10–19 age range.

Five studies included individuals exclusively 15 years and younger [25,45,47,61,81], three of which included individuals exclusively 13 years and younger [25,45,81]. Nineteen studies included individuals exclusively aged 14 years and older [22,23,27,28,29,31,37,40,43,46,51,56,58,59,63,68,73,78,79], six of which included individuals exclusively aged 16 years and older [23,29,31,58,63,78]. Thirty-four studies provided an age range including both individuals aged 13 years and younger as well as individuals aged 16 years and older [21,26,30,32,33,34,35,36,38,41,42,44,49,50,52,53,54,55,57,60,62,64,65,66,67,69,70,71,72,74,75,76,77,80]. A full breakdown of participant age range included in each study can be found in Supplementary Table S2A.

### 3.3. Country of Publication

The country of publication of the original research studies included the United States (*n* = 21, 34.4%) [27,31,32,33,39,46,48,49,53,56,57,58,60,61,63,66,69,72,77,78,81], the United Kingdom/Sweden (*n* = 6 each, 11.8%) [21,22,23,29,40,45,50,54,55,67,76,80], Netherlands/Australia (*n* = 4 each, 7.8%) [24,26,30,44,64,71,74,75], Canada (*n* = 3, 5.9%) [51,52,68], Norway/South Africa/Romania/Brazil/Multi-country (*n* = 2 each, 3.9%) [25,28,34,35,37,38,42,65,73,79], and North India/Italy/Poland/Israel/Iran/Portugal/Kuwait (*n* = 1 each, 1.6%) [36,41,43,47,59,62,70].

### 3.4. Ethnicity/Ancestry of Participants

Of the 61 original research and dissertation studies, 23 studies provided information about ethnicity/ancestry/race. Nineteen of these studies provided numbers and/or percentages of the self-reported ethnic/ancestral groups included in the studies [27,31,32,33,39,40,46,49,50,53,56,57,58,60,63,69,71,72,80]. Of these 19 studies, 13 reported that the majority (>50%) of individuals identified as white/white non-Hispanic/white British/European American. African American/black individuals were represented in 15 of these studies. Hispanic/Latino/Spanish individuals were represented in 10 of these studies. Eight studies included individuals who were Asian/south Asian. One study included individuals who identified as Native American or Arab.

### 3.5. Socioeconomic Status of Participants

Eleven studies included individuals’ socioeconomic status. Five included individuals predominately from a low socioeconomic status/economically disadvantaged area [39,41,43,72,81], while two studies were conducted in low to moderate/middle socioeconomic status areas [28,78]. Two studies represented a mix of low, medium/middle, and high socioeconomic status [35,61]. One study included individuals mostly from the middle and middle upper class [31], and one study provided the mean household income for individuals [49].

### 3.6. Gender/Sex of Participants

Fifty-five studies included information about the gender/sex of the participants. Six studies included over 70% male participants [24,26,32,33,49,67], while eleven studies included over 70% female participants [45,46,47,50,54,55,56,63,65,68,69]. Of the eleven female-dominated studies, three studies did not include any males (100% female) [45,56,63]. There were no studies that included only males. One study included five non-binary individuals [60].

### 3.7. Type of Conditions/Disabilities

**Figure 3:**
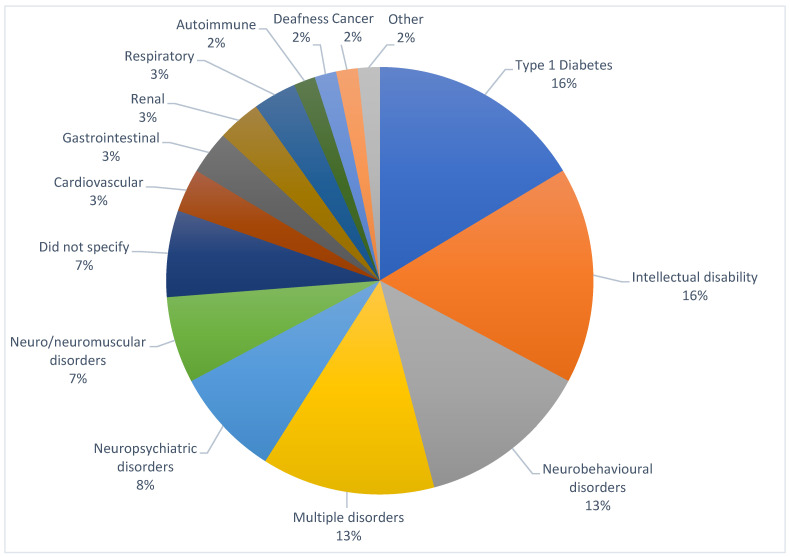
Types of conditions/disabilities represented in studies.

The types of conditions/disabilities represented in the studies included type 1 diabetes (*n* = 10, 16%) [21,36,38,52,57,59,65,70,73,79], intellectual disability (*n* = 10, 16%) [30,34,35,43,49,56,58,74,75,77], neurobehavioral disorders (*n* = 8, 13%) [24,27,28,32,33,39,61,78], multiple disorders (*n* = 8, 13%) [41,51,55,62,66,68,76,80], neuropsychiatric disorders (*n* = 5, 8%) [31,40,53,64,67], neuro/neuromuscular disorders (*n* = 4, 7%) [37,44,47,48], did not specify (*n* = 4, 7%) [25,29,50,72], cardiovascular (*n* = 2, 3%) [22,23], gastrointestinal (*n* = 2, 3%) [26,45], renal (*n* = 2, 3%) [42,71], respiratory (*n* = 2, 3%) [46,81], autoimmune (*n* = 1, 2%) [69], deafness (*n* = 1, 2%) [60], cancer (*n* = 1, 2%) [54], and other (*n* = 1, 2%) [63] (Figure 3).

The studies represented in the intellectual disability (ID) category included participants with ID, as well as participants with ID or learning disabilities (LD). Neurobehavioral disorders included those with autism spectrum disorders (ASDs), attention deficit hyperactivity disorder, LD, and/or those who require special accommodations/education. Neuropsychiatric disorders included depression, generalized anxiety disorder, psychosis, bipolar disorder, eating disorders, and/or mood disorders. Neuro/neuromuscular disorders included cerebral palsy, epilepsy, spastic diplegia, spastic hemiplegia, spinal muscular atrophy, and/or muscular dystrophy. Additional disorders were divided into categories based on organ systems (e.g., congenital heart disease was categorized as cardiovascular). Studies with multiple conditions falling into more than one category were placed in the ‘multiple’ section (e.g., studies including both multifactorial and congenital anomalies were placed in the multiple section). One study focusing on individuals with inherited bleeding disorders was placed in the ‘other’ category, as it did not fit in any other categories. A full breakdown of each condition/disability included in each study can be found in Supplementary Table S2.

## 4. Interventions

Of the 61 original research studies, 30 (49% of studies) administered/designed some form of an intervention within the population to improve empowerment or empowerment-adjacent elements (i.e., self-efficacy, shared decision making, etc.). Empowerment was measured as an outcome following all interventions.

The interventions were administered/designed in an in-person/group format (*n* = 20, 66.7%), or an online format (*n* = 8, 26.7%). Two interventions were placed in the ‘other’ category, as they were not in-person/group or online interventions (*n* = 2, 6.7%). Seven of the studies administering/designing an intervention compared results between an intervention and non-intervention group (control), and four of these studies determined that empowerment was higher in the intervention groups compared to the control. Twenty-three studies did not include a control group; however, seven of these studies compared empowerment levels at pre- and post-intervention stages. In six of these studies, empowerment levels were reported to have increased after the intervention. In fifteen of the studies without a control group, participants reported that the intervention was empowering (Table 1).

### 4.1. Tools Used to Measure Empowerment

In total, 27 studies (44.3%) utilized a quantitative study design with various tools including scales and questionnaires to measure empowerment, while 26 studies (42.6%) utilized a qualitative study design with various tools including interviews, focus groups, and journal entries to measure empowerment. Qualitative data were analyzed using thematic coding analysis for all studies. Eight studies (13.1%) utilized a mixed-methods study design to measure empowerment. In total, the most common quantitative tools used to measure empowerment were the ARC-Self-determination scale (SDS) (*n* = 11) and the Gothenburg Young Person’s Empowerment Scale (GYPES) (*n* = 6). The most common qualitative tools were interviews (*n* = 31) and focus groups (*n* = 8) (Table 2).

### 4.2. Domains and Outcomes Associated with Empowerment

Several articles identified domains and outcomes associated with empowerment. These were identified in quantitative studies as having a correlation with empowerment levels in various scales or questionnaires and were identified in qualitative studies as common themes emerging from interviews/focus groups associated with empowerment.

These outcomes included self-control (*n* = 14) [27,30,32,33,40,50,52,58,59,68,70,77,79,81], self-determination (*n* = 12) [27,32,33,34,35,37,49,56,58,60,68,78], self-confidence/esteem/worth/concept/expression (*n* = 12) [24,25,35,45,48,49,51,57,61,68,75,80], a sense of belonging/community/support/involvement (*n* = 12) [28,30,31,40,41,45,49,53,61,69,76,81], shared decision making/participation (*n* = 11) [25,29,31,40,41,42,50,54,55,65,76], self-efficacy (*n* = 8) [32,33,35,48,68,69,77], self-management/treatment/care (*n* = 7) [21,31,38,53,71,73,79], autonomy (*n* = 7) [27,35,51,68,71,73,76], increased knowledge/skills/information (*n* = 7) [29,31,38,48,54,70,73], independence (*n* = 5) [24,26,39,43,76], self-advocacy (*n* = 4) [27,56,61,73], higher quality of life (*n* = 4) [30,39,43,81], receiving a diagnosis/owning one’s disability label (*n* = 4) [31,35,61,81], and improved treatment adherence (*n* = 3) [36,46,69].

### 4.3. Review Articles

Six review articles were included in this scoping review. Three focused on understanding the impact of interventions in young individuals with chronic disease/long-term conditions. These included mental health [84], mobile health [85], and patient education [86] interventions, all of which identified empowerment as an outcome. Two other studies identified empowerment as a theme in young individuals with epilepsy [83] and cystic fibrosis [3]. One review focused on patient family-centered care in young individuals with various chronic conditions and found empowerment as an outcome associated with engagement and collaborative care [82].

## 5. Discussion

This is the first scoping review to identify evidence of empowerment in adolescents with a disability/chronic condition. We will highlight topics including countries of publication, demographic variables, types of conditions, interventions described (including the involvement of adolescents as research partners), domains associated with empowerment, and finally, instruments used to measure empowerment. The results of this scoping review indicated that there were a greater number of studies including participants over the age of 16 compared those including participants under the age of 13. Two studies reported higher levels of empowerment in older individuals compared to younger individuals [22,44]; however, one study found that empowerment levels in individuals with diabetes decreased with increased duration of disease (i.e., older individuals) [59]. This contradiction signifies the importance of future research in this field, including a large age range of adolescent perspectives to gain insight into different levels of empowerment observed in younger compared to older adolescents.

The high proportion of US-based studies included in this review (34.4%) was consistent with reviews similar in nature when empowerment and other similar themes (e.g., self-efficacy) were evaluated. Four of the six review articles included in this scoping review reported that over 40% of included studies were published in the US [3,84,85,86]. In our review, the two most common disabilities/chronic conditions included were type 1 diabetes and intellectual disability (each 16%). However, these disabilities/chronic conditions have been reported as having lower incidence/prevalence rates in the US compared to other countries [87,88]. Population-based studies have determined that intellectual disability in children/adolescents is more common in low- and middle-income countries (i.e., India, Pakistan, Bangladesh, etc.) [87], and epidemiological data have determined that Northern Europe has the highest rate of type 1 diabetes [88]. Given the contrast in the rates of these conditions compared to the geographical location where the research took place (primarily in the US), we emphasize the importance of this research in countries outside of the US to capture the lived experiences of a diverse range of individuals.

Ethnicity/ancestry, socioeconomic status, and gender/sex of participants were reported in less than half of the studies included in this review, and few studies included significant findings of empowerment compared between these demographic groups. One study found that psychological empowerment and community participation was partially mediated by the socioeconomic status of participants [37]. Additionally, lower quality of life has been reported in children with chronic disease in lower socio-economic backgrounds [89]. This signifies an important gap in research where demographic characteristics are not being considered or consistently observed, as they may represent critical findings. A review aiming to explore the lived experiences of ethnic minority youth with disabilities highlighted this importance and identified themes within this population such as environmental and systemic barriers in navigating healthcare. The authors also indicated the importance of the intersectionality of disability, ethnicity, socioeconomic status, and gender/sex [90].

Our review included a broad range of disabilities/chronic conditions to generate common themes across the literature and across varying conditions. It was found that many studies including a wide variety of conditions administered interventions and reported outcomes related to empowerment. Some interventions were administered in an online format, which was common in studies aiming to promote self-management/treatment/care in young individuals with type 1 diabetes [21,36,38,79]. Self-management of diabetes and glycemic control has been reviewed extensively in the literature [91], though less frequently in the adolescent population. It has been reported that self-management of diabetes is effective in increasing glycemic control; however, work needs to be carried out in evaluating the psychosocial outcomes of self-management in children and adolescents [91]. Our review identified studies demonstrating high levels of empowerment following interventions to improve self-management of diabetes [21,36,38,79]; however other mental health implications such as depression and diabetes distress should also be considered [91]. One study included in our review identified that individuals ranged from feeling empowered to feeling fearful in self-management of their diabetes [52]. Additionally, it is important to recognize the role that socioeconomic factors play in the self-management of diabetes to best understand how to develop and integrate the appropriate tools necessary to support different populations. This gap is evident in our review where these factors are not widely considered, and these considerations have been discussed specifically within the type 1 diabetes population [92].

Interventions also commonly occurred within the ID, neurobehavioral, and neuropsychiatric disorder populations, where many studies reported an increase in empowerment levels post-intervention [24,27,56,58,61,64,75]. Although some studies without interventions identified a general trend of high empowerment levels within these populations, others reported low empowerment levels, specifically in individuals with ID [30,43,74,77] or ASD [33]. One study identified that individuals with ID had higher empowerment scores when receiving parental and classroom support, highlighting the importance of support resources within this population [49]. A systematic review and meta-analysis compiling data on interventions used to promote self-determination in individuals with ID highlighted the need for further research to help facilitate supportive environments to help meet the psychological needs of this population [93].

Another theme amongst the interventions identified in this review was the use of adolescents as research partners leading to feelings of empowerment [25,29,41,73,81]. A scoping review on adolescents as co-researchers was conducted in 2021 and found similar results of increased empowerment, increased knowledge and skills, and increased self-esteem [94]. Based on these findings, in conjunction with our review, we suggest that strategies and interventions used to promote empowerment or outcomes related to empowerment consider involving adolescents as collaborators or partners in research. However, this review also noted the gap in our understanding of the ethical implications stemming from involving young individuals in research as well as time consumption and funding issues [94]. Although the findings from studies included in our review were primarily positive, it is important to consider these additional factors when conducting studies including adolescent research partners.

Of the included domains associated with empowerment identified in this review, self-control was observed most frequently. In another review, self-control along with self-efficacy, self-management, and increased knowledge of condition were all observed as outcomes associated with psycho-educational interventions for children with chronic disease [95]. These themes were all found to be linked to empowerment in our scoping review. It has also been found that implementation of a family-centered empowerment program in adolescents with thalassemia major resulted in a significant increase in self-efficacy [96]. In a systematic review and meta-ethnography study, empowerment was described in a conceptual model to understand what aspects of mental health interventions children and young people with long-term physical conditions found most important. This study found that empowerment was facilitated via self-esteem and was linked to increased social support [84]. Our review also identified the importance of self-esteem and social support/community involvement in promoting empowerment. We suggest that future research continue to develop, implement, and observe empowerment interventions within the adolescent population of disabled individuals and focus on further evaluation of these key outcomes. Psychological empowerment programs have also been shown to decrease stress, anxiety, and depression in adolescents with hemophilia [97]. This provides additional support to the importance of empowerment interventions and their impact on the wellbeing of this population.

The studies included in our review were nearly evenly distributed between quantitative and qualitative study designs, representing a diverse range of data collection. The most commonly used quantitative tool was the ARC-SDS, which is not surprising given that many studies represented individuals with ID or neurobehavioral disorders, where this tool has been frequently used [93]. The GYPES was also commonly used and has been established as a validated tool used to measure empowerment in young people [98]. This tool consists of five domains: knowledge and understanding, personal control, identity, decision making, and enabling others. The GYPES has been validated using confirmatory factor analysis and is appropriate in evaluating empowerment in individuals with any chronic condition [98]. Future research aiming to evaluate empowerment in adolescents with any chronic condition may consider using this scale to better understand empowerment levels across individuals with varying conditions.

Interviews and focus groups were conducted in both qualitative and mixed-method studies and were able to provide several emerging empowerment-related themes. It is critical that research within the adolescent population continue to qualitatively access the lived experiences of those with disabilities/chronic conditions, as the information collected in interviews and focus groups offers additional context to quantitative data. Effective methods and barriers in qualitative research of children/adolescents have been reviewed and interview techniques such as building appropriate rapport have been suggested in the literature [99]. One study in our review conducted interviews as well as allowing participants to complete journal entries to share their feelings about living with chronic kidney disease [71]. Journal entries have been used in other studies as starting points alongside interviews in younger adolescents (under 14 years old) with spina bifida where children were given the opportunity to create a unique journal about their experiences [100]. This suggests an important tool which future studies aiming to understand the lived experiences of adolescents could consider in addition to the use of interviews and focus groups. This may be especially beneficial in younger adolescents, providing them a place to begin expressing their feelings.

### 5.1. Limitations

Our review was limited to studies published in English and accessible through UBC library, resulting in the potential for low representation of areas where English is not the primary language and exclusion of studies not available to the authors through the library database. More than half of the studies did not include detailed demographic information about their participants (i.e., ethnicity/ancestry and socioeconomic status). This missing information must be considered when interpreting our demographic findings.

Some studies evaluating interventions lacked control groups, and other studies provided very low sample sizes (*n* < 4). Findings generated from these studies must be carefully interpreted. Additionally, the term ‘empowerment’ was defined differently by various studies in this review. Although there were many overlapping themes, it must be considered that studies may have been measuring different domains of the term ‘empowerment’.

### 5.2. Conclusions

This scoping review identified a gap in the consideration of ethnicity/ancestry and socioeconomic status in adolescents with a chronic condition/disability in studies evaluating empowerment. We also identified a gap in the inclusion of younger adolescents in this research space and suggest that future research should aim to capture the experiences of a broad range of adolescent ages. Additionally, several interventions were observed to have positive outcomes on empowerment levels in adolescents with a chronic condition/disability. Evaluating these interventions specifically in cohorts underrepresented in this scoping review (i.e., in countries outside of the US, lower-socioeconomic-status groups, ethnic minority groups, and younger adolescents) is needed to fully capture their outcomes and broader applications.

We suggest that future research focus on understanding the intersection of disability, ethnicity, and socioeconomic status in adolescents with a chronic condition/disability and how best to implement interventions used to increase empowerment in various demographic settings.

## Figures and Tables

**Table 1:** Intervention summaries and outcomes.

Goal of Intervention	Length of Intervention	Control Group	Empowerment Related Outcome(s)	Ref.
**In-person/group activities:** **School curriculum (*n* = 8)**				
Self-advocacy instruction to educate students about their disability and develop self-advocacy skills	One-quarter-long high school class	No	Participants reported higher empowerment levels after intervention (compared to baseline)	[27]
School-to-work internship program designed to provide students with functional and vocational post-secondary skills	Full-year program	Yes	Intervention did not show significant increase in empowerment compared to control group	[39]
Paths to the future (P2F) program to promote self-efficacy/awareness, disability knowledge, and gender identity	75 lessons divided into 4 modules	No	Participants reported higher empowerment levels after intervention (compared to baseline)	[56]
Peers Engaged in Effective Relationships-Decision Making (PEER-DM) to promote decision making, self-determination/evaluation/advocacy, and goal setting	45 min class once per week over six weeks	Yes	Higher empowerment was reported in intervention group compared to control	[58]
Eye to Eye program organized by mentoring students with LD teaches social–emotional learning strategies through an art-based curriculum	One-year after-school program + 5-day summer program	No	Participants reported feeling empowered through the intervention	[61]
TAKE CHARGE intervention based on a multi-component self-determination model designed to increase motivation and self-efficacy including mentorship for adolescents	One semester (weekly 50 min coaching sessions)	No	Participants reported higher empowerment levels after intervention (compared to baseline)	[66]
Project-based learning (PBL) instructional approach to engage students to collaborate and initiate critical thinking/reflection	One semester (every other day)	No	Participants reported feeling empowered after the intervention	[68]
Choice maker curriculum enables students to learn self-determination skills and achieve their goals	4-week intervention	No	Overall reported increase in self-determination, but empowerment did not specifically increase as a sub-domain of self-determination (compared to baseline)	[78]
**In-person/group activities:** **Adolescents as research partners/collaborators** **(*n* = 5)**				
Leadership program where group leaders designed sessions to provide adolescents with more initiative overtime and eventually develop a research theme and conduct a small-scale research project	8 meetings over 4 months	No	Participants reported feeling empowered as research partners	[25]
Co-led participatory research project with disabled participants to develop a research agenda to address a quality- and rights-based approach for planning futures	15-month research project	No	Participants identified the need to feel engaged in the research project to promote empowerment	[29]
Community-based rehabilitation program to promote engagement and collaboration where participants help to organize studies	6 dissemination sessions	No	Participants reported that group-centered rehabilitation led to group empowerment	[41]
Young leaders in diabetes learning knowledge and skills to manage their condition and are encouraged to develop new projects at the end of training	One-semester-long training	No	Participants moved up the empowerment ladder (felt more empowered) after training	[73]
SMART program to improve asthma knowledge formed through a collaborative research process where students are partners and integrate their own perspectives	Twice a week for 20 weeks	No	Participants reported higher empowerment levels after intervention (compared to baseline)	[81]
**In-person/group activities:** **Community-based programs** **(*n* = 3)**				
KONTAKT Social skills group training sessions promoting social interaction/communication skills, problem solving, and confidence	12–24 sessions (60–90 min per week)	No	61% of participants reported feeling more empowered after the intervention	[24]
Residential immersive life skills (RILS) program takes place in college residences to build communication skills	3 weeks	Yes	Participants reported a significant increase in psychological empowerment 3 months after intervention and compared to control	[51]
Pilot structured education program designed to include knowledge about diabetes pathophysiology to enable adolescents with T1D to develop self-management skills	3 days	No	Participants reported that the intervention led to knowledge and empowerment	[70]
**In-person/group activities:** **Therapeutic program** **(*n* = 2)**				
Expressive music therapy in group sessions to achieve therapeutic change	16 sessions (held weekly)	No	Participants reported that the intervention was an empowering experience	[64]
Therapeutic recreation summer camp helps campers realize their abilities and develop coping strategies, resilience, and independence	5-day residential activity camps	No	Participants reported higher empowerment levels after intervention (compared to baseline)	[80]
**In-person/group activities:** **Physical activities (*n* = 2)**				
Dance and yoga intervention called TIME (Try, Identify, Move, and Enjoy) built on the foundation of self-determination theory	Twice weekly for eight months (30 min of dance, 25 min of yoga, 5 min of reflection)	No	Participants reported that the intervention led to feelings of empowerment	[45]
Power soccer using motorized wheelchairs to promote self-efficacy and communication	Athletes had been playing for up to 10 years	No	Participants reported that playing power soccer leads to empowerment and independence	[48]
**Online:** **Diabetes self-management (*n* = 4)**				
Continuous glucose monitoring (CGM) + ehealth care program with nurses providing advice through texts/calls to help set glycemic control goals with patients	2-week program repeated after 3, 6, and 12 months (option to continue if more support was needed)	Yes	Participants reported a significant increase in empowerment in intervention compared to control group	[21]
Serious game play for diabetes health designed to enhance knowledge and management of diabetes while also providing entertainment	6 training sessions (2 h each) followed by 20 min of gameplay	No	Participants reported feeling empowered after playing the game	[36]
Mobile phone-based visualization tool to understand food intake including a food diary and diabetes message system to promote knowledge and self-management	3 full days (at least 2 days were mandatory)	No	Participants reported feeling empowered after using the tool	[38]
CGM to allow participants to measure their glucose levels in real time and help to manage their diabetes	Participants had been using CGM for at least 3 months	No	Participants reported that using CGM was empowering	[79]
**Online:** **Educational programs** **(*n* = 2)**				
Online education program about Systemic Lupus Erythematosus (SLE) where feedback and questions were encouraged from participants	8 modules over 8 weeks	Yes	Higher empowerment was reported in intervention group compared to control	[69]
The Growth Factory (TGF) online intervention providing a structured learning environment for youth with ID focusing on key growth mindset affirmations	6 sessions over 6 weeks (25–45 min each)	Yes	Intervention did not show significant increase in empowerment compared to control group	[75]
**Online:** **Development of tools with participants (*n* = 2)**				
An educational video intervention was co-designed with participants to improve youth question asking	Single interview	No	Participants reported that being involved in research and designing the video was empowering	[65]
Ideas gained from a formative study to help develop a digital decision support tool to help increase adolescent participation, self-efficacy, and autonomy	Single interview	No	Participants reported feeling empowered through involvement and support	[76]
**Other (*n* = 2)**				
Transanal irrigation (TAI) used to treat fecal inconsistent and constipation and improve adherence, independence, and quality of life	1 month and 6-month follow-up after treatment	No	Participants reported empowerment levels after intervention (consistent with empowerment levels seen in patients with other types of conditions)	[26]
Skills for growing up epilepsy communication tool addressing knowledge and skills required for self-management and autonomy in daily functioning	Tool used once	Yes	No difference in empowerment levels reported between intervention and control groups	[44]

**Table 2:** Summary of tools used to measure empowerment.

Study Design	Tool Used to Measure Empowerment	Ref(s).
**Quantitative (*n* = 27)**	ARC-SDS: psychological empowerment measured as a sub-domain (*n* = 7)	[32,33,34,35,37,58,78]
	GYPES (*n* = 5)	[21,22,23,26,46]
	Diabetes Empowerment Scale (DES) (*n* = 3)	[36,57,59]
	Quality of Life (QOL) questionnaire (*n* = 2)	[39,43]
	Dutch questionnaire: EMPO youth 2.0 (*n* = 2)	[74,75]
	Quality of Student Life questionnaire (QSLQ) (*n* = 1)	[30]
	ASL Self-determination inventory (*n* = 1)	[60]
	Family Empowerment Scale (FES) (*n* = 1)	[66]
	Validated Empowerment Likert Scale (*n* = 1)	[69]
	Health self-empowerment theory variables (*n* = 1)	[72]
	Young Leaders in Diabetes (YLD) training test scores (pre- and post- intervention) (*n* = 1)	[73]
	Adult Nowicki–Strickland Internal–External Scale (ANS-IE), Intellectual Achievement Responsibility Questionnaire (IARQ), Self-efficacy and Outcome Expectancy Scale (*n* = 1)	[77]
	Sociopolitical Control Scale for youth (*n* = 1)	[81]
**Qualitative (*n* = 26)**	Interviews (*n* = 20)	[24,28,29,31,38,40,45,47,48,50,52,53,55,63,67,68,70,76,79,80]
	Interviews + focus groups (*n* = 4)	[25,41,61,65]
	Focus groups (*n* = 1)	[42]
	Interviews + journal entries (*n* = 1)	[71]
**Mixed methods (*n* = 8)**	Interviews and/or focus groups + ARC SDS (*n* = 4)	[27,49,51,56]
	Focus groups + GYPES (*n* = 1)	[44]
	Interviews + integrative literature review (*n* = 1)	[54]
	Interviews + focus groups + self-report questionnaires (*n* = 1)	[62]
	Interviews + feedback questionnaires (*n* = 1)	[64]

## Supplementary Materials

The following supporting information can be downloaded at: https://www.mdpi.com/article/10.3390/children12010049/s1, Table S1A: Search Strategy; Table S1B: Data Extraction; Table S2A: Summary of Original Articles; Table S2B: Summary of Review Articles.
