# Supplementary material for: Empowerment in Adolescent Patients with a Disability/Chronic Condition: A Scoping Review
Source: Children (Basel). 2024 Dec 31;12(1):49. doi: 10.3390/children12010049 (PMC11764063; doi:10.3390/children12010049)
Supplement: Supplementary file 1 [file children-12-00049-s001.zip › Supplementary Tables.pdf]

Supplementary Table S1. Search Strategy and Data Extraction

A) Search strategy

NO.	KEYWORDS
1.	Chronic Disease/
2.	(disorder* or disease* or condition* or disabilit*).mp.
3.	empowerment/
4.	empower*.mp.
5.	Pediatrics/
6.	Adolescent/
7.	Child/
8.	Young Adult/
9.	(young adult* or young people or child* or adolescen* or youth or pediatric* or high school or middle school).mp.
10.	5 or 6 or 7 or 8 or 9
11.	1 or 2
12.	3 or 4
13	5 or 6 or 7 or 8 or 9
14.	11 and 12 and 13

Table S1A. The MEDLINE search strategy. The star symbol (*) denotes unlimited truncation, allowing the search to include word variants formed from different suffixes. The symbol .mp

refers to words that appear in different fields, including title, abstract, subject heading, author keywords, while subject headings are indicated by the slash (/) suffix. Row 14 represents the set of citations forwarded for screening.

B) Data extraction

NO.	DATA EXTRACTION CATEGORIES
**1**	Study characteristics (reference ID, first author, article title, year of publication, journal name, type of publication)
**2**	Population or sample characteristics (age range, country of publication, ethnicity/ancestry, socioeconomic status, gender/sex, type of disability/chronic condition)
**3**	Setting (use of intervention)
**4**	Data collection tools (study design, qualitative methods, quantitative methods, control group)
**5**	Outcomes (empowerment-related outcomes and additional outcomes associated with empowerment)
**6**	Summary (study objective and outcomes)

Table S1B. Data extracted from included publications for Supplementary Table S2 and Discussion.

First Author	Year	Age range	Type of condition/ disability	Objective(s)	Empowerment related outcome(s)
Abebe	2023	13 - 16	Type 1 diabetes (T1D)	To investigate the effect of a novel eHealth care program on empowerment and metabolic control for adolescents with T1D.	The Gothenburg Young Person's Empowerment Sale (GYPES) scores of adolescents in the intervention group showed a statistically significant increase over time.
Acuna Mora	2019	14 - 18	Congenital heart disease	To determine empowerment levels and examine their correlations in youth with congenital heart disease.	Empowerment levels varied across the group. Higher levels of patient empowerment were associated with older age and improved quality of life.
Acuna Mora	2022	16 - 18	Congenital heart disease	To determine the association between patient empowerment and different patient reported outcomes in youth with congenital heart disease.	Youth with higher empowerment levels across time reported better communication skills than those with lower empowerment levels.
Afsharnejad	2022	Mean = 13.6 $\pm$ 1.7 (LR) = 13.7 $\pm$ 1.6 (HR)	Autism spectrum disorder (ASD)	To understand the lived experiences of autistic adolescents and their parents after receiving the KONTAKT ® social skills group training program.	Perceived improvements in empowerment-related skills due to the KONTAKT program included independence, self-confidence, and factors of emotionality.
Andersen	2023	9 - 12	Did not specify	To understand how participatory methods in research with disabled children contribute to empowerment.	Findings demonstrated that participatory methods can be effective tools in the empowerment process of developing a child's initiative, self-confidence, and agency.
Baaleman	2022	10 - 16	Gastro-intestinal disorders	To investigate the patient experience and clinical effect of Transanal irrigation (TAI) in children, baseline data was compared to one and six-month follow ups.	Most children (61%) reported slightly or greatly increased independence after TAI. Patient empowerment, measured using GYPES, showed a median total empowerment score of 55.
Beaulieu	2007	14 - 18	Special education	To explore the efficacy of self-advocacy instruction on secondary special	A significant increase in psychological empowerment was seen

				education students with disabilities.	between pre- and post-instruction scores.
Botha	2023	15 - 17	ADHD	To explore illness perception in adolescents with ADHD using semi-structured interviews.	One theme that emerged from interviews was ‘self-empowerment strategies’, with subthemes ‘controlling external stimuli’, ‘accepting support from others’ and ‘personalized learning strategies’.
Brady	2019	16 - 23	Did not specify	To conduct a research study co-led by six disabled young people and address ethical issues that were identified in this partnership.	The young researchers became empowered throughout the project, but also brought tensions surrounding this compared to personal experiences where the youth were given few opportunities for empowerment.
Bramston	2002	12 - 18	Intellectual disability (ID)	To understand how sense of community connectiveness is related to perceived quality of life in adolescents with and without ID.	Participants with ID reported significantly lower empowerment/control than those without, as determined by a subscale of the Quality of Student Life Questionnaire.
Bringewatt	2014	18 - 22	Depression, anxiety & bipolar disorder	To understand how children make sense of their mental health disorder diagnosis and how diagnostic labels can be best communicated to children.	It was found that children experienced both empowerment and stigma associated with their diagnoses. Children were empowered by their diagnosis and by open communication.
Chou	2014	13 - 21	ASD, ID, Learning disability (LD)	To evaluate the differences in self-determination in students with ASD, ID, and LD.	Students with ASD, ID, and LD varied in psychological empowerment levels.
Chou	2016	13 - 22	ASD, ID, LD	To evaluate the differences in self-determination in students with ASD, ID, and LD.	Students with LD had the highest levels of psychological empowerment, which were significantly higher than those with ASD.
Cristea	2023	13 - 16	ID, LD	To explore the relationship patterns and influences of educational and familial environments on students’ disability	The results do not suggest a mediating role between opportunities at school and psychological empowerment (a sub-

				labels and self-determination.	domain of self-determination).
Cristea	2023	12 - 19	ID, LD	To examine the degree to which different family and personal factors affect self-concept and self-determination in students with ID and LD.	Self-concept was found to predict psychological empowerment. The family environment configuration also showed a role in predicting empowerment.
D'Aprile	2019	11 - 17	T1D	To assess the relationship between serious game play, patient empowerment, diabetes management, and gender differences using a theoretical framework.	Game experience was found to significantly predict diabetes empowerment, and patient empowerment significantly positively influenced adherence-related behaviours of diabetes.
Figueiredo	2023	15 – 32*	Cerebral Palsy	To determine whether family socioeconomic status and self-determination mediates the relationship between the mobility and community involvement of adolescents with Cerebral Palsy.	The association between mobility and breadth of community participation was mediated indirectly by psychological empowerment and was moderated by socioeconomic status.
Frøisland	2015	13 - 19	T1D	To implement a tool used to capture the food intake of adolescents with T1D into care and to evaluate the effect of the tool on empowerment, self-efficacy and self-treatment.	Participants reported feeling empowered after using the tool.
Goldstein	2013	Mean = 19.2 ± 1.32	Special needs	To examine the impact of a School-to-Work Internship Pilot Program designed for high school seniors with disabilities.	No statistical difference in empowerment was found between the program participants and control group.
Grealish	2013	14 - 18	Psychosis	To conceptualize empowerment from the experiences and perceptions of young people experiencing psychosis.	Six main empowerment-related themes were identified through interviews: Individual control and choice vs inflexibility; being listened to, respected, and validated; communication; response of services; coping and

					structure; and quality of relationship and support.
Gulati	2011	12 - 18	Multiple	To understand how adolescents with disabilities can take more control over their rehabilitation and involvement in a community-based rehabilitation program.	The Adolescent Group Empowerment Pyramid conceptual framework was developed including four central themes; group participation, demonstration, recognition and interactions between the socio-cultural environment and disability.
Gutman	2018	8 - 21	Chronic kidney disease	To explore the perspectives of children with chronic kidney disease and their parents on decision making and communication.	Adolescents identified feeling disempowered when they felt a lack of knowledge and understanding, and when there was poor communication with clinicians.
Hematian	2009	14 - 26	ID	To understand the differences between individuals with and without ID on their perspectives about their quality of life.	Adolescents without ID had higher scores of empowerment/ independence compared to individuals with ID.
Hilberink	2018	8 - 19	Epilepsy	To assess the impact of epilepsy on empowerment and autonomy in youth and evaluate the feasibility of using the Skills for Growing Up (SGU)-Epilepsy communication tool in this population.	Older youth with epilepsy appeared to have higher empowerment scores compared to their younger counterparts. Use of the SGU-Epilepsy tool showed no effect on empowerment.
Högström	2023	9 - 13	Functional abdominal pain disorders	To assess the impact of Just in TIME (Try, Identify, Move, and Enjoy), a dance and yoga intervention, on the experiences of adolescent females with functional abdominal pain disorders.	Girls that participated in the Just in TIME intervention felt empowered in several ways, including ability to manage pain and stress, speaking up for themselves and others, and creating new social connections.
Holder	2022	14 - 23	Cystic Fibrosis	To examine the association between empowerment and transition readiness, treatment adherence, and health-related	Empowerment was found to have a positive association with treatment adherence and transition readiness. Empowerment showed no

				quality of life in adolescents with Cystic Fibrosis.	association with health-related quality of life.
Hutzler	2002	9 - 15	Various neuro-muscular	To understand the experiences of children with physical disabilities in physical education, and to identify both supporting and limiting factors towards their empowerment and inclusion.	No general trend was found between inclusion and empowerment. Over half of the children speaking of experiencing failure in physical education supported the idea of empowerment.
Jeffress	2017	10 – 52*	Various neuro-muscular	To explore the experiences and perceived benefits/opportunities of power soccer in individuals with physical disabilities.	Power soccer provided opportunity to the players that was a major source of empowerment and improved self-efficacy.
Jones	2009	11 - 20	ID	To better understand the personal experiences of adolescents with ID and the association of global self-worth with other factors.	Adolescent perception of parental support was positively correlated with psychological empowerment. Students in resource rooms had significantly higher empowerment scores than students in self-contained classrooms.
Jordan	2019	13 - 19	Did not specify	To explore adolescents' experiences and perspectives on what they see as barriers and facilitators to their involvement in shared decision-making.	'Sense of empowerment' was found to be a theme surrounding involvement in shared decision-making.
King	2023	14 - 21	Multiple	To assess the association between self-determination changes and Residential Immersive Life Skills program participation in youth with disabilities.	There were no significant changes over time in empowerment for either the intervention group or the control group.
Koller	2014	5 – 18*	T1D	To explore children's and adolescents' perspectives of self-care, knowledge about diabetes, and emotions related to their diabetes diagnosis.	The emotional impact of the knowledge of diabetes was found to range from fear to empowerment.

Kranke	2013	12 - 17	Depression, bipolar disorder & ADHD	To understand the experiences and perceptions of adolescents undergoing psychiatric treatment to determine conditions that promote empowerment.	Individuals with less self-stigma and subsequent feelings of empowerment had four common overlapping themes – supportive family/friends, school success, positive outlook, and involvement in treatment.
Lea	2018	13 - 24	Cancer	To better understand adolescent experiences of care and create an evidence-based, operational model defining age-appropriate care for young adults with cancer.	'Empowering young people' was found to be one of the fundamental components of age-appropriate care. Adolescents felt they should be informed of their current situation and treatment options as well as being involved in decision-making.
Lewis	2020	11 - 18	Multiple	To explore young peoples' understandings, motivations, concerns, and experiences with decision-making regarding genome sequencing.	Young people that were asked to sign an assent form saw it as empowering, allowing them a choice in whether to participate.
Lindstrom	2018	14 - 20	ID, LD	To determine the impact and outcomes of the Paths 2 the Future curriculum through the perspectives of young women with disabilities.	'Empowerment and voice' was a theme that came up in the focus group. The curriculum allowed the young women to develop skills and confidence to 'speak up' and stand up for themselves.
Lu	2014	13 - 25	T1D	To understand attitudes around the use of peer mentoring as an intervention for promoting adherence in adolescents with T1D.	Adolescents had high empowerment scores on the diabetes empowerment scale.
Luber	2018	16 - 19	ID	To assess the effectiveness of the Peers Engaged in Effective Relationships-Decision Making (PEER-DM) instructional tool in improving self-	A significant increase in psychological empowerment (a sub-domain of self-determination) scores was seen in the intervention group compared to the control group.

				determination in adolescents with ID.	
Luczynski	2019	14 - 18	T1D	To determine whether readiness to change and empowerment is related to the clinical features and executive function of adolescents with Type 1 Diabetes.	Diabetes empowerment was found to decrease with increasing duration of the disease and increased with poorer metabolic control, as this deficiency encouraged readiness to change.
Millen	2021	13 - 22	Deaf or hard of hearing	To explore self-determination scores in youth who are deaf or hard of hearing to determine if scores vary by communication type, disability, or educational environment.	Psychological empowerment was the subdomain of self-determination with the highest overall mean score.
O'Heaney	2018	10 - 15	LD	To explore how the mentoring program ‘Eye to Eye’ uses social-emotional learning strategies to improve empowerment and self-esteem in students with LD.	‘Feeling empowered and owning your label’ was a subtheme that was identified, along with additional empowerment-adjacent themes such as self-esteem and self-advocacy.
Pais	2019	12+	Multiple	To understand the experience of chronic disease in family, education, and hospital settings, and to identify predictors and models of wellbeing in children with chronic diseases.	Both interactional and self-centered behavioural empowerment in children were found to be associated with their wellbeing.
Parker	2022	16 - 18	Inherited bleeding disorders	To understand the experiences of adolescents related to managing heavy menstrual bleeding after receiving a bleeding disorder diagnosis.	Participants often felt empowered by their diagnosis, as it allowed for identity formation, reduced amount of fear, and increased knowledge.
Patterson	2015	13 - 17	Eating disorders, mood disorders, anxiety & psychotic disorders	To assess the feasibility and impact of implementing a music therapy program in psychiatric wards for adolescent patients.	‘Empowering’ was a descriptor used by participants when expressing their feedback about the program, along with ‘relaxing’, ‘comforting’, and ‘uplifting’.
Pembroke	2021	11 - 17	T1D	To co-design a video intervention approach	Participants suggested the integration of a positive

				with youth with T1D to improve youth question-asking.	message into the video intervention to empower youth.
Powers	2001	12 - 18	Multiple	To evaluate the impact of the TAKE CHARGE model on psychosocial adjustment, disability-related self-efficacy, empowerment, and personal accomplishment in youth with physical disabilities.	Significant improvements in empowerment, psychosocial adjustment, and level of adolescent accomplishment were seen post-intervention, and were at significantly higher levels than the control group.
Ringer	2023	10 - 18	PANS	To better understand the experience and perspectives of children living with PANS.	Children experienced empowerment when treatments were flexible and adapted to their needs specifically.
Robertson	2015	14 - 18	Multiple	To identify and assess the factors influencing the implementation and design of a project-based learning curriculum within a public alternative education program from student and staff perspectives.	A theme across students was empowerment through self-determination, self-expression, and self-expression, where they noted higher levels of autonomy and recognition of their strengths.
Scalzi	2018	13 -23	SLE	To assess the feasibility and effects of a web-based educational program pilot on self-management in adolescents and young adults with SLE.	The intervention group showed higher mean scores on all empowerment outcomes compared to the control group.
Taha	2020	11 - 16	T1D	To explore the social and psychological impact of a pilot small-education group for diabetes self-management on youth with T1D and their mothers.	The education program empowered participants by increasing their knowledge and subsequent feelings of control over their diabetes.
Tong	2013	12 - 24	Chronic kidney disease	To understand the experiences and perspectives of youth with chronic kidney disease waiting for a kidney transplant.	Empowerment, autonomy, and maturity were identified as subthemes of ‘resilience’ in interviews with the participants.
Tucker	2014	12 - 17	Did not specify	To examine the impact of Health Self-	The Health Self-Empowerment Theory

				Empowerment Theory on predicting levels of engagement in health-promoting behaviours in low-income chronically ill adolescents.	combined variables explained 30-38% of the variance in the different health-promoting behaviours.
Ugliara Barone	2021	15 - 25	T1D	To describe the Empowerment Ladder stages and process in youth before and after completing the Youth Leaders in Diabetes Training program.	Post training test scores were significantly higher than baseline pre-training scores, indicating that youth had progressed along the Empowerment Ladder.
Verberg	2019	12 - 23	ID	To assess relationships between mindset and perseverance, empowerment, mental health problems, and self-esteem in youth with and without ID.	Significant positive correlations were found between perseverance and both empowerment and self-esteem. Youth with ID had lower levels of empowerment than youth without ID.
Verberg	2022	12 - 23	ID	To evaluate the effectiveness of The Growth Factory, an online mindset intervention, for youth with ID.	No significant effects of the intervention were seen in empowerment measures.
Vinblad	2019	6 - 17	Multiple	To explore the perspectives and experiences of youth with disabilities to create a support tool to increase participation of children in pediatric rehabilitation.	An overall theme extracted was ‘moving toward empowerment of children in pediatric rehabilitation’, which was found to relate to youth feeling involved, feeling independent, and working in partnership.
Wehmeyer	1994	13 - 20	LD, ID	To explore psychological empowerment in youth with LD and ID.	Students with LD scored significantly lower in the outcome expectancy measure of psychological empowerment than students without LD.
White-Hector	2013	17 - 21	Various neuro-behavioural	To investigate if the ChoiceMaker curriculum improves self-determination in high school students with disabilities.	There was no statistical difference in empowerment scores before and after the intervention.
Williams	2023	14 - 16	T1D	To assess illness perceptions in a group of adolescents with T1D	Adolescents reported that CGM increased their sense of control over their

				using continuous glucose monitoring (CGM).	diabetes management, which empowered them to take responsibility of their care and increased their self-confidence.
Yang	2022	10 - 18	Multiple	To assess the experiences of children attending a therapeutic recreation summer camp to understand its impact on their self-esteem, social functioning, wellbeing, and health challenges.	Participants felt that the camp created a sense of empowerment and improved their self-confidence. Some adolescents felt more confident and empowered about managing their condition.
Yarbrough	2016	12 - 13	Asthma	To determine the impact of the Student Media-based Asthma Research Team (SMART) school program on students with asthma, their peers, and their parents.	Students showed significant improvements in sociopolitical empowerment, emotional support, asthma control, and asthma-related quality of life during the intervention period.

Supplementary Table S2A: Summary of original papers included in the review

**First Author**	**Year**	**Age Range**	**Number of papers**	**Objective(s)**	**Empowerment related findings**
Allen	2018	16-25	24	A systematic review and qualitative meta-synthesis to review the literature on patient- and family-centered care (PFCC) for young adults with chronic diseases.	One of the main elements of PFCC was that patients and families felt empowered to be involved in their care. Young adults wanted to feel engaged in collaborative care, and not a passive recipient. Poor communication was found to disempower patients and their families.
Chong	2016	3-21	43	A systematic review of qualitative studies to describe the experiences of children and adolescents with epilepsy.	Young people reported feeling empowered when they were provided with support and relevant information about epilepsy, treatment, and broader psychosocial impacts. They reported feeling empowered as they were able to accept and control their disease.
Fairweather	2022	6-21	17	A meta-synthesis of qualitative literature	While the individual studies included did not explicitly

				surrounding empowerment in children and youth with cystic fibrosis.	speak about empowerment, the authors generated common themes around facilitators of, barriers to, and components of empowerment.
Shaw	2019	≤ 25	60	A systematic review and meta-ethnography conducted to understand what aspects of mental health and wellbeing-focused interventions that children and young people with long term physical conditions saw as most important.	Empowerment was one of five identified constructs in the review and relevant quotations from youth were provided. In a conceptual model, it was shown that empowerment (via skills and knowledge, self-esteem, and mastery) was interlinked with a therapeutic foundation, social support, and a hopeful alternative.
Slater	2017	15-24	12	A systematic review of current qualitative evidence on young adults' experiences of using mobile(m)-Health technologies for chronic disease management.	Empowerment was one of the subthemes identified under 'perceptions of benefits' in using the mHealth technology interventions. Young adults felt that the technologies improved their knowledge and skills for self-management, which increased confidence and empowerment.
Stenberg	2019	3-29 (mean = 12.14)	69	A scoping review of the potential impact of patient education interventions on young adults, adolescents, and children with chronic illnesses and/or impairment loss.	Self-efficacy/Empowerment was identified as a common outcome, which was measured using various quantitative scales.

Supplementary Table S2B: Summary of reviews included in the paper

LR = Low responders, HR = High responders

*Adolescent ages (10-19) were separated with results

PANS- Pediatric Acute Onset Neuro-psychiatric Syndrome

SLE- Systemic Lupus Erythematosus
